# Quantitative Analysis of Prostate MRI: Correlation between Contrast-Enhanced Magnetic Resonance Fingerprinting and Dynamic Contrast-Enhanced MRI Parameters

**DOI:** 10.3390/curroncol30120750

**Published:** 2023-12-04

**Authors:** Moon-Hyung Choi, Young-Joon Lee, Dongyeob Han, Dong-Hyun Kim

**Affiliations:** 1 Department of Radiology, Eunpyeong St. Mary’s Hospital, College of Medicine, The Catholic University of Korea, Seoul 03312, Republic of Korea; cmh@catholic.ac.kr; 2 Siemens Healthineers Ltd., Seoul 06620, Republic of Korea; dongyeob.han@siemens-healthineers.com; 3 School of Electrical and Electronic Engineering, Yonsei University, Seoul 03722, Republic of Korea; donghyunkim@yonsei.ac.kr

**Keywords:** magnetic resonance fingerprinting, dynamic contrast-enhanced magnetic resonance imaging, quantitative analysis, prostate, prostate neoplasm

## Abstract

This research aimed to assess the relationship between contrast-enhanced (CE) magnetic resonance fingerprinting (MRF) values and dynamic contrast-enhanced (DCE) MRI parameters including (K^trans^, K_ep_, V_e_, and iAUC). To evaluate the correlation between the MRF-derived values (T1 and T2 values, CE T1 and T2 values, T1 and T2 change) and DCE-MRI parameters and the differences in the parameters between prostate cancer and noncancer lesions in 68 patients, two radiologists independently drew regions-of-interest (ROIs) at the focal prostate lesions. Prostate cancer was identified in 75% (51/68) of patients. The CE T2 value was significantly lower in prostate cancer than in noncancer lesions in the peripheral zone and transition zone. K^trans^, K_ep_, and iAUC were significantly higher in prostate cancer than noncancer lesions in the peripheral zone (*p* < 0.05), but not in the transition zone. The CE T1 value was significantly correlated with K^trans^, V_e_, and iAUC in prostate cancer, and the CE T2 value was correlated to V_e_ in noncancer. Some CE MRF values are different between prostate cancer and noncancer tissues and correlate with DCE-MRI parameters. Prostate cancer and noncancer tissues may have different characteristics regarding contrast enhancement.

## 1. Introduction

The Prostate Imaging—Reporting and Data System (PI-RADS) recommends a multiparametric prostate MRI protocol that encompasses T2-weighted imaging (T2WI), T1-weighted imaging (T1WI), diffusion-weighted imaging (DWI), and dynamic contrast-enhanced (DCE) MR [1]. As a contrast material and additional scan time are necessary to obtain DCE MRI, the usefulness of biparametric MRI without DEC MRI has been proven [2,3,4]. However, DCE MRI helps to diagnose clinically significant prostate cancer in the peripheral zone that shows early enhancement, especially in the case where DWI is degraded [5]. PI-RADS recommends qualitatively reviewing DCE MRI and determining whether early enhancement is present in the focal lesion. Although visual analysis based on the relative signal intensity of the lesion compared to the surrounding normal tissue is the common way to interpret DCE MRI, research on quantitative analyses has continued to produce objective parameters [6].

DCE MRI, which repeatedly obtains many images on the same section with a very short time interval, provides a change in signal intensity in the pixel over time. PI-RADS version 1 suggested interpreting DCE MRI by classifying time-intensity curves among three types [7]. Some studies have shown a high proportion of type 3 curves (rapid enhancement and washout) in prostate cancer [8,9]. However, curve type analysis showed poor performance in differentiating prostate cancer from healthy tissue [10]. Moreover, quantitative DCE parameters (K^trans^, K_ep_, V_e_, and iAUC) from the Tofts model have been suggested to explain the pharmacokinetic characteristics of contrast material in prostate cancer [11]. K^trans^, K_ep_, and iAUC are higher in prostate cancer than in benign or normal tissue and are higher in more aggressive cancer than in less aggressive cancer, especially in the peripheral zone [9,12,13,14,15].

In terms of quantitative analysis, magnetic resonance fingerprinting (MRF) has emerged as a method to measure multiple tissue properties with relatively shorter scan times than conventional mapping methods [16]. MRF-derived T1 or T2 values were significantly lower in prostate cancer than in noncancer or benign tissue [17,18,19]. In some research, MRF was acquired before and after contrast enhancement, and the T1 value significantly decreased on contrast-enhanced (CE) MRF compared with nonenhanced (NE) MRF [20]. The contrast-enhanced T1 and T2 values exhibited significant differences when comparing prostate cancer to normal tissue [21]. However, the CE MRF-derived T1 and T2 values of prostate cancer were not explored in the peripheral zone and transition zone separately. Additionally, CE MRF values and DCE parameters, the quantitative parameters related to CE MRI, may be correlated. The purpose of this study was to evaluate the correlation between CE MRF values and DCE-MRI parameters (K^trans^, K_ep_, V_e_, and iAUC) as well as to validate the difference in the parameters between prostate cancer and noncancer lesions.

## 2. Materials and Methods

The institutional review board of the hospital approved this study, and the requirement for informed consent was waived owing to its retrospective design.

### 2.1. Patients

**Figure 1:**
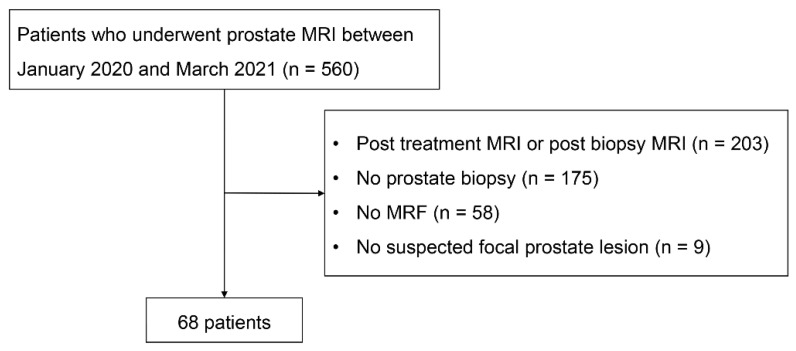
Flowchart of the patient inclusion process.

We searched all prostate MRI examinations performed in our institution between January 2020 and March 2021. Among 560 examinations, MRI examinations were excluded according to the following criteria: (1) patients with known prostate cancer including post-treatment or postbiopsy status (n = 203); (2) patients who did not undergo prostate biopsy (n = 198); (3) MRI examinations without MRF (n = 77); (4) patients without suspected prostate cancer (PI-RADS ≥ 3) (n = 9); and (5) DCE MRI was not obtained (n = 5). A total of 68 patients who underwent prostate prebiopsy MRI and prostate biopsy for prostate focal lesions were included in this study (Figure 1).

Patient clinical information, including age and prostate-specific antigen (PSA) level before prostate MRI, was collected. Location and PI-RADS v.2.1 classification were recorded from the MRI reports that were already generated during the clinical process by one of two abdominal/genitourinary radiologists with more than 10 years of experience. If the prostate focal lesion involved both the peripheral zone and the transition zone, the location of the lesion was determined by the center of the lesion. One of the two radiologists performed transrectal ultrasound-guided prostate biopsy using an ultrasound-MRI fusion system (Logiq E10, GE healthcare, Chicago, IL, USA). The radiologist performed targeted and systematic biopsy and reported the location of the targeted biopsy. Pathology results were reported by one of four board-certified pathologists and included the Gleason grade group of prostate cancer, which was defined by the International Society of Urological Pathology, and the number and location of positive cores.

### 2.2. MRI Protocol

The study involved all patients undergoing multiparametric MRI without an endorectal coil on a 3-T system (Magnetom Vida, Siemens Healthineers, Erlangen, Germany), utilizing a 30-channel body coil along with either a 32-channel or 72-channel spine coil. The specific MRI parameters are detailed in Table 1. Dynamic contrast-enhanced (DCE) MRI was conducted using the Golden-angle RAdial Sparse Parallel (GRASP) technique, with a temporal resolution of 4.3 s for the first 17 s, followed by 7 s for the subsequent 180 s. To calculate the DCE MRI parameters, T1 maps were generated through the variable flip angle technique, employing angles of 2° and 15°. MRF was conducted twice, first before the injection of contrast material and then immediately following the completion of DCE MRI.

MRF has been integrated into the prebiopsy prostate MRI protocol for patients for patients with the clinical suspicion of prostate cancer. MRF data were obtained using a hybrid radial/echo-planar imaging (EPI) trajectory [19]. This involved a golden-angle rotating radial acquisition in the kxy domain, combined with simultaneous EPI acquisition in the slice encoding direction (kz), employing a sinusoidal flip angle to achieve high-resolution MRF data. The scan time of each MRF was 3 min 48 s. For both NE and CE MRF, the same parameters were used: sinusoidal 320 flip angles, TR = 16 ms, TE = 4 ms, resolution = 0.6 × 0.6 × 3 mm^3^, FOV = 160 × 160 × 72 mm^3^ and scan time = 3 min 48 s. The dictionary was generated based on the Bloch equation in MATLAB (The MathWorks, Natick, MA). The T1 range of the dictionary was 50 msec to 3000 msec with a 10-msec step size and was 3050 msec to 4000 msec with a 50-msec step size. The T2 range of the dictionary was 5 msec to 250 msec with a 1-msec step size, 252 msec to 350 msec with a 2-msec step size, and 355 msec to 400 msec with a 5-msec step size. Dictionary matching using the inner-product method was performed to acquire quantitative T1 and T2 maps from NE and CE MRF.

### 2.3. Image Analysis

Two radiologists with 10 and 23 years of experience (each having read over 1000 cases) independently analyzed the DCE MRI and MRF images without access to each others’ results. We used commercial software (Syngo.via VB70B, Siemens Healthineers) to analyze DCE MRI and open-source software (ITK-SNAP version 3.8.0 [www.itksnap.org [accessed on 30 November 2023]]) to analyze the MRF maps. Four parameters were calculated from the DCE MRI: K^trans^ (volume transfer constant that represents the leakage of contrast from the vascular to the extravascular component); V_e_ (fractional volume of extravascular extracellular space [EES] per unit tissue volume); K_ep_ (reflux rate constant that describes contrast reflux from the EES back into the vascular component); and the initial area under the time-to-signal intensity curve (iAUC) measured during the first 60 s [22]. T1 and T2 values were acquired from the NE MRF maps, and CE T1 and CE T2 values were acquired from the CE MRF maps. We calculated T1 change (%) and T2 change (%) as [(NE value − CE value)/NE value] × 100.

**Figure 2:**
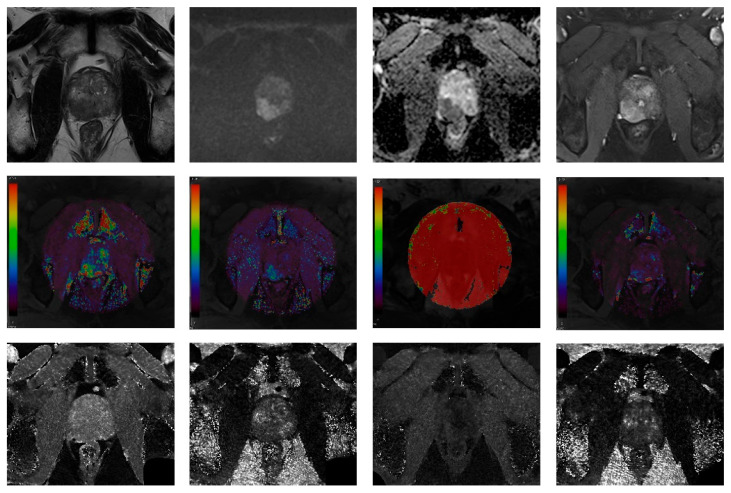
A 66-year-old patient with elevated prostate-specific antigen (15.5 ng/mL).

Rough information about the location of the most severe prostate focal lesion (right or left, peripheral zone or transition zone) that was reported in the clinical MRI report was provided to the radiologists. However, they were unaware of the biopsy results. They independently drew a region-of-interest (ROI) covering the abnormal focal lesion on the axial image that contained the largest diameter of the lesion. The average values of the parameters were extracted from the ROIs. The images of a representative case are presented in Figure 2.

A 2.5 cm hypointense bulging lesion was detected in the right peripheral zone on the T2-weighted image (T2WI) with a high signal intensity on the B = 1500 mm^2^/s diffusion-weighted image (DWI), a low value on the apparent diffusion coefficient (ADC) map, and early enhancement on the dynamic contrast-enhanced (DCE) MRI (T2WI, DWI, ADC map and DCE MRI in order from left, top row). The DCE parametric maps (K^trans^, K_ep_, V_e_, and iAUC in order, middle row) and MRF maps (nonenhanced [NE] T1, NE T2, contrast-enhanced [CE] T1, and CE T2 maps in order, bottom row) are presented.

### 2.4. Statistical Analysis

The characteristics of the patients were summarized by presenting the mean and standard deviation for continuous variables, and the frequency and percentage for categorical variables.

Inter-reader agreement for all image parameters was assessed with an intraclass correlation coefficient (ICC). The ICC was interpreted as <0.5, poor; 0.5–0.75, moderate; 0.75–0.9, good; and >0.9, excellent agreement.

Prostate focal lesions were classified into prostate cancer and noncancer according to the biopsy results. We compared the clinical characteristics and quantitative parameters between prostate cancer and noncancer by lesions using Student’s *t* tests. The analysis of the correlations between the DCE MRI parameters and MRF values was conducted through Pearson’s correlation tests. For statistical analysis, SPSS software version 24.0 (IBM, Armonk, NY, USA) and GraphPad Prism version 8.0 (GraphPad Software, Inc., La Jolla, CA, USA) were used. A *p* value below 0.05 was regarded as statistically significant.

## 3. Results

Table 2 shows the characteristics of the patients. The mean PSA of the patients was 37.7 ± 112.2 ng/mL. The prostate focal lesion was located in the peripheral zone in 46 patients (67.6%). More than half of the lesions (51.5%) were classified as PI-RADS 5. Prostate cancer was diagnosed in 51 patients (75.0%), and prostate cancer with Gleason grade group 1 was diagnosed in 4 patients.

Inter-reader agreement for the CE T1 values and the iAUC was excellent; that for the T2 change was moderate; and that for the rest of the variables was good (Table 3).

Patients with prostate cancer were significantly older than patients without prostate cancer (71.8 ± 9.2 versus 64.6 ± 10.8, *p* = 0.009), but the PSA level was not different between the two groups. The PI-RADS classification was higher in prostate cancer (4.63 ± 0.49) than noncancer lesions (4.12 ± 0.49) (*p* = 0.001).

Table 4 shows the differences in the image parameters between noncancer and prostate cancer in the peripheral zone for reader 1 and reader 2. For both readers, the CE T2 values were significantly lower in prostate cancer than in noncancer. The T1 and T2 values were lower in prostate cancer than in noncancer without statistical significance. Among the DCE parameters, K^trans^, K_ep_, and iAUC were significantly higher in prostate cancer than in noncancer. In the transition zone, only the CE T2 value was significantly lower in prostate cancer than in noncancer for both readers, although the CE T1 value was significantly higher in prostate cancer than in noncancer by reader 2 (Table 5). No DCE parameter was significantly different between cancer and noncancer.

**Figure 3:**
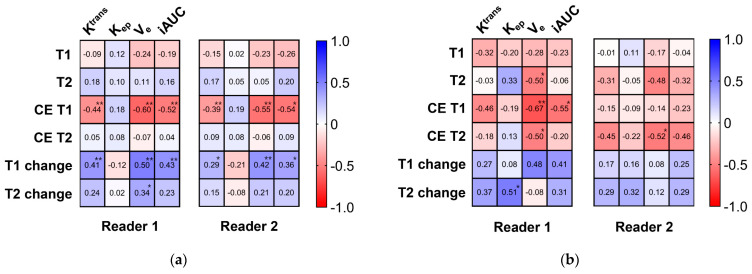
Correlation matrix between MR fingerprinting and DCE MRI parameters. Correlation matrices between MR fingerprinting parameters (T1 value, T2 value, CE T1 value, CE T2 value, T1 change, and T2 change) and DCE MRI parameters (K^trans^, K_ep_, V_e_, and iAUC) are presented for reader 1’s result (left) and reader 2’s results (right) in cancer (**a**) and noncancer (**b**). * *p* < 0.05, ** *p* < 0.01.

**Figure 4:**
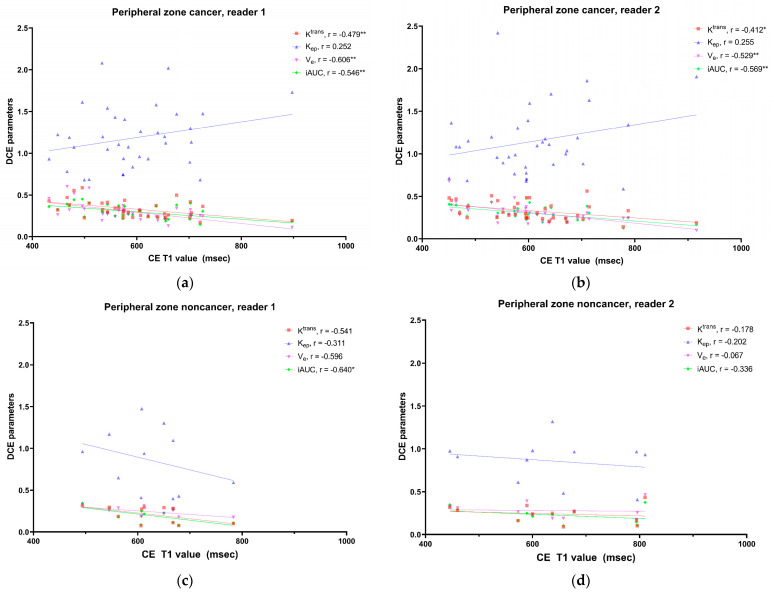
Correlation between CE T1 map and DCE parameters in peripheral zone lesions. Scatter plots show the correlation between the CE T1 value and DCE parameters in prostate cancer and noncancer for reader 1 (**a**,**c**) and reader 2 (**b**,**d**). * *p* < 0.05, ** *p* < 0.01.

Correlation coefficients between the MRF parameters and DCE MRI parameters are presented in Figure 3. In prostate cancer, the CE T1 value was negatively correlated, and the T1 change was positively correlated with three DCE parameters (K^trans^, V_e_, and iAUC). The T1 value was not correlated with any DCE parameters. In noncancer, a significant correlation was commonly noted only between the CE T2 value and Ve for both readers. The correlation between the CE T1 value and DCE parameters in the peripheral zone lesions is presented in Figure 4. The CE T1 value was significantly correlated with K^trans^, V_e_, and iAUC in prostate cancer. In contrast, no DCE parameter showed a significant correlation with the CE T1 value in noncancer.

## 4. Discussion

In this study, we acquired NE MRF and CE MRF as well as DCE MRI as a part of prebiopsy prostate MRI in patients who underwent prostate biopsy. We evaluated the parameters from MRF to DCE MRI in prostate focal lesions with PI-RADS classification ≥ 3, and the lesions were divided into prostate cancer and noncancer according to the biopsy results. Therefore, we could evaluate the differences in the MRI parameters between prostate cancer and noncancer lesions in the peripheral zone and the transition zone. Additionally, we investigated the correlations amongst the T1 and T2 values from NE MRF and CE MRF with the DCE MRI parameters in prostate cancer and noncancer lesions.

Among DCE parameters, Ve was not different between prostate cancer and noncancer lesions. K^trans^, K_ep_, and iAUC were significantly higher in prostate cancer than in noncancer lesions in the peripheral zone but not in the transition zone. Our study agreed with many studies that showed similar results. A previous study showed that K^trans^, K_ep_, and AUC for 90 s after injection were significantly higher in prostate cancer than in the normal peripheral zone or in prostate cancer than in benign lesions [14,23]. Other studies showed that K^trans^ and iAUC were significantly higher in low-grade cancer than in high-grade cancer in the peripheral zone, but not in the transition zone [7,13]. No DCE parameter was significantly different between tumor and benign nodules in the transition zone [24]. Ve was not different between prostate cancer and benign tissue or between clinically significant cancer and clinically insignificant cancer in the studies [14,25,26].

We evaluated T1 and T2 values from NE and CE MRF values and the changes in T1 and T2 values. The differences in MRF-derived CE T1 and CE T2 values between prostate focal lesions (including cancer) and the normal peripheral zone or transition zone have been reported previously [20,21]. The differences in CE MRF values between noncancer and prostate cancer have not been evaluated. For both readers, only the CE T2 value was significantly lower in prostate cancer than in noncancer lesions in the peripheral zone and transition zone. As no study has evaluated CE T1 or T2 values between prostate cancer and noncancer, a further evaluation is necessary to validate the results. We interpret this result based on the same context as a previous study; the CE T2 value was significantly lower in prostate cancer than the normal peripheral zone or transition zone [21]. However, the CE T1 value was not significantly different between cancer and noncancer lesions. These results were not expected because we usually use T1WI to evaluate CE MRI. Obtaining CE MRF more than 3 min after contrast injection may be the reason why the CE T1 value was not different between prostate cancer and noncancer. The absolute T1 value of the focal lesion in the delayed phase may not be related to the enhancement pattern in the early phase when prostate cancer commonly shows early enhancement and rapid washout [10,27]. The amount of contrast that is retained in prostate cancer may not be enough to make a significant difference compared to noncancer in the delayed phase.

Regarding NE MRF, both readers’ results commonly showed that T1 and T2 values were not different between prostate cancer and noncancer lesions in the peripheral zone and transition zone. Some previous studies showed significantly lower T1 and T2 values in prostate cancer than in noncancer lesions in both the peripheral zone and transition zone [17,18]. In another study that analyzed prostate focal lesions regardless of location, the T2 value was significantly lower in prostate cancer with Gleason grade group ≥ 2 than in noncancer. In the current study, the T2 value of peripheral zone cancer was lower than that of noncancer, but it did not reach statistical significance. Additionally, there was no significant difference in the T1 and T2 values between prostate cancer and noncancer lesions in the transition zone. The differences in the results between the current study and previous studies may be due to the small number of patients in each zone and the noncancer group in this study.

Correlations between CE MRF values and DCE parameters have not been evaluated. The CE T1 value was negatively correlated with K^trans^, V_e_, and iAUC in prostate cancer but not in noncancer lesions. The same results were observed in the peripheral zone. As K^trans^ represents the permeability of the vasculature, a higher K^trans^ means a larger amount of contrast leakage into the EES that causes a greater decrease in the T1 value. Among DCE MRI parameters, V_e_ showed the strongest correlation with the CE T1 value in prostate cancer. Tissue with a larger V_e_, which is the fractional volume of the EES per unit tissue volume, may retain more contrast material, leading to a greater reduction in the T1 value on CE MRF. We noted that K_ep_, which describes contrast reflux from the EES back into the vascular component, was not correlated with any MRF parameters in prostate cancer and noncancer. CE MRF was acquired after DCE MRI and may represent the characteristics of the tissue in the delayed phase. Therefore, K_ep_ may not affect the CE T1 value in the delayed phase. Thus, this study proved that CE MRF values have different meanings from the DCE parameters; this is understandable because CE MRF values are absolute values measured at a certain time point and DCE parameters are used to measure the pharmacokinetic effects of the contrast material.

There are several limitations in this study. First, this study is a retrospective study. Although all patients underwent MRI-ultrasound fusion biopsy, it was difficult to assure that the focal lesion on prebiopsy MRI was successfully targeted. However, we believe that MRI-ultrasound fusion biopsy is the best clinically available method. Second, the number of patients, especially the number of patients with transition zone lesions, was relatively small. This study evaluated patients who underwent prebiopsy CE MRI, including NE and CE MRF and biopsy. The inclusion criteria for this study were stringent, resulting in a limited number of participants. Despite this constraint on sample size, the findings offer valuable insights. Future research with larger patient cohorts will be pivotal in confirming and enhancing the understanding gained from this study. Third, image analysis was performed on a single axial image that may not reflect the characteristics of the entire lesion. The high inter-reader agreement between the two readers in this study may compensate for the limitation of the single-section image analysis. However, it is noteworthy that despite the high inter-reader agreement between the two readers, the significance of the statistical analyses showed slight variations between them. This could be attributed to the relatively small size of prostate lesions, as including a slightly different number of pixels could lead to minor yet significant alterations in the measurements. Fourth, while statistically significant correlations were identified between MRF-derived values and DCE parameters, these findings did not hold sufficient clinical relevance to routinely substitute DCE-MRI with CE MRF from a quantitative standpoint. Nonetheless, considering that correlations between DCE parameters and MRF values have not been explored previously, presenting these results appears to be meaningful.

## 5. Conclusions

Some CE MRF values are different between prostate cancer and noncancer tissues and correlated with DCE-MRI parameters. Prostate cancer and noncancer tissues may have different characteristics regarding contrast enhancement.

## Figures and Tables

**Table 1:** MRI parameters.

	Axial T2WI (TSE)	Coronal T2WI (TSE)	Sagittal T2WI (TSE)	3D T2WI (CS 3D SPACE)	T1WI (TSE)	DWI	MRF	DCE MRI (GRASP)
TR (ms)	2500–3000	2510	4220	1800	500–700	5200–6000	16	4
TE (ms)	103	103	100	104	9	76	4	2
Field of views (mm)	180 × 180	180 × 180	180 × 180	200 × 200	180 × 180	200 × 180	160 × 160	200 × 200
Matrix	320 × 320	640 × 640	640 × 640	577 × 577	320 × 320	120 × 108	256 × 256	224 × 224
Resolution (mm)	0.6 × 0.6	0.3 × 0.3	0.3 × 0.3	0.3 × 0.3	0.6 × 0.6	1.7 × 1.7	0.6 × 0.6	0.9 × 0.9
Flip angle (degrees)	136	136	120	135	120	90	Sinusoidal flip angle	12
Slice thickness (mm)	3	3	3	0.6	3	3	3	3
Gap	0	0	0	0	0	0	0	0
NEX	2	2	6	2	2	2, 2, 9, 9	1	1
B values (s/mm^2^)	-	-	-	-	-	0, 100, 1000, 1500	-	-
Acquisition time (min:s)	3:32	3:17	3:17	4:55	3:35	5:58	3:48	3:41

TR, repetition time; TE, echo time; T2WI, T2-weighted image; TSE, Turbo spin echo; CS, compressed sensing; SPACE, sampling perfection with application-optimized contrasts using different flip angle evolution; T1WI, T1-weighted image; DWI, diffusion-weighted image; MRF, magnetic resonance fingerprinting; DCE MRI, dynamic contrast-enhanced MRI; GRASP, Golden-angle RAdial Sparse Parallel MRI; NEX, number of excitations.

**Table 2:** Baseline characteristics of patients.

Characteristics	Value
Number of patients	68
Age (years)	70.0 ± 10.0
PSA (ng/mL)	37.7 ± 112.2
PI-RADS classification (*n* [%])	
3	1 (1.5)
4	32 (47.1)
5	35 (51.5)
Location of the focal lesion (*n*, [%])	
Peripheral zone	46 (67.6)
Transition zone	22 (32.4)
Prostate cancer (*n*, [%])	51 (75.0)
Peripheral zone	35 (68.6)
Transition zone	16 (31.4)
Gleason grade group (*n*, [%])	
1	4 (5.9)
2	14 (20.6)
3	18 (26.5)
4	10 (14.7)
5	5 (7.4)

PSA, prostate-specific antigen; PI-RADS, Prostate Imaging—Reporting and Data System.

**Table 3:** Inter-reader agreement in MRF and DCE parameters.

Parameters	ICC	*p* Value
MRF		
T1 value (,)	0.825	<0.001
T2 value (ms)	0.898	<0.001
CE T1 value (ms)	0.968	<0.001
CE T2 value (ms)	0.859	<0.001
T1 change (%)	0.892	<0.001
T2 change (%)	0.708	<0.001
DCE MRI		
K^trans^	0.870	<0.001
K_ep_	0.854	<0.001
V_e_	0.854	<0.001
iAUC	0.904	<0.001

ICC, intraclass correlation coefficient; ADC, apparent diffusion coefficient; MRF, magnetic resonance fingerprinting; CE, contrast-enhanced; DCE MRI, dynamic contrast-enhanced magnetic resonance imaging; iAUC, initial area under the curve.

**Table 4:** Differences in parameters between cancer and noncancer in peripheral zone.

Parameters	Reader 1			Reader 2		
	Noncancer (n = 11)	Prostate Cancer (n = 35)	*p* Value	Noncancer (n = 11)	Prostate Cancer (n = 35)	*p* Value
MRF						
T1 value (ms)	1626.1 ± 171.1	1488.2 ± 137.0	0.009	1622.4 ± 169.5	1489.7 ± 210.9	0.064
T2 value (ms)	99.6 ± 27.7	83.1 ± 12.6	0.081	95.17 ± 22.84	79.62 ± 11.70	0.051
CE T1 value (ms)	624.9 ± 77.7	593.3 ± 96.7	0.330	640.1 ± 125.6	608.4 ± 101.2	0.397
CE T2 value (ms)	95.04 ± 26.58	74.9 ± 11.2	0.032	96.41 ± 26.55	71.93 ± 10.51	0.019
T1 change (%)	61.41 ± 4.29	60.0 ± 6.34	0.491	60.76 ± 4.85	58.83 ± 6.79	0.386
T2 change (%)	3.999 ± 8.771	9.424 ± 7.349	0.047	−1.935 ± 16.863	6.904 ± 5.786	0.116
DCE MRI						
K^trans^	0.209 ± 0.100	0.332 ± 0.101	0.001	0.241 ± 0.104	0.328 ± 0.107	0.021
K_ep_	0.857 ± 0.382	1.181 ± 0.358	0.013	0.857 ± 0.260	1.146 ± 0.402	0.031
V_e_	0.242 ± 0.056	0.298 ± 0.107	0.105	0.279 ± 0.089	0.307 ± 0.122	0.489
iAUC	0.196 ± 0.059	0.301 ± 0.078	0.001	0.227 ± 0.091	0.299 ± 0.084	0.018

ADC, apparent diffusion coefficient; MRF, magnetic resonance fingerprinting; CE, contrast-enhanced; DCE MRI, dynamic contrast-enhanced magnetic resonance imaging; iAUC, initial area under the curve.

**Table 5:** Differences in parameters between cancer and noncancer in transition zone.

Parameters	Reader 1			Reader 2		
	Noncancer (n = 6)	Prostate Cancer (n = 16)	*p* Value	Noncancer (n = 6)	Prostate Cancer (n = 16)	*p* Value
MRF						
T1 value (ms)	1596.0 ± 115.1	1575.8 ± 143.7	0.762	1608.9 ± 141.8	1547.9 ± 188.7	0.483
T2 value (ms)	81.77 ± 15.22	76.61 ± 7.93	0.306	76.32 ± 11.44	74.19 ± 9.52	0.662
CE T1 value (ms)	519.87 ± 72.06	591.62 ± 94.44	0.109	490.1 ± 48.8	607.5 ± 103.8	0.016
CE T2 value (ms)	80.32 ± 9.94	70.02 ± 8.13	0.021	72.02 ± 5.56	65.00 ± 7.37	0.048
T1 change (%)	67.15 ± 6.05	62.48 ± 4.67	0.068	69.32 ± 4.29	60.79 ± 4.26	<0.001
T2 change (%)	0.003 ± 14.261	8.629 ± 4.679	0.202	4.107 ± 14.595	12.138 ± 4.957	0.061
DCE MRI						
K^trans^	0.372 ± 0.165	0.376 ± 0.139	0.954	0.322 ± 0.173	0.384 ± 0.118	0.342
K_ep_	1.042 ± 0.464	1.247 ± 0.372	0.292	0.998 ± 0.527	1.313 ± 0.420	0.159
V_e_	0.356 ± 0.063	0.306 ± 0.079	0.178	0.318 ± 0.055	0.302 ± 0.076	0.650
iAUC	0.320 ± 0.137	0.340 ± 0.116	0.730	0.286 ± 0.154	0.343 ± 0.100	0.317

ADC, apparent diffusion coefficient; MRF, magnetic resonance fingerprinting; CE, contrast-enhanced; DCE MRI, dynamic contrast-enhanced magnetic resonance imaging; iAUC, initial area under the curve.

## Data Availability

The datasets generated or analyzed during the study are not publicly available due to privacy protection for patients but are available from the corresponding author on reasonable request.
